# Chest-wall reconstruction with a customized titanium-alloy prosthesis fabricated by 3D printing and rapid prototyping

**DOI:** 10.1186/s13019-017-0692-3

**Published:** 2018-01-08

**Authors:** Xiaopeng Wen, Shan Gao, Jinteng Feng, Shuo Li, Rui Gao, Guangjian Zhang

**Affiliations:** Department of Thoracic Surgery, First Affiliated Hospital of Xi’an Jiaotong University, #277 West Yanta Road, Xi’an, Shaanxi Province 710061 People’s Republic of China

**Keywords:** 3D printing, Titanium-alloy prosthesis, Chest-wall bony defect, Rapid prototyping

## Abstract

**Background:**

As 3D printing technology emerge, there is increasing demand for a more customizable implant in the repair of chest-wall bony defects. This article aims to present a custom design and fabrication method for repairing bony defects of the chest wall following tumour resection, which utilizes three-dimensional (3D) printing and rapid-prototyping technology.

**Methods:**

A 3D model of the bony defect was generated after acquiring helical CT data. A customized prosthesis was then designed using computer-aided design (CAD) and mirroring technology, and fabricated using titanium-alloy powder. The mechanical properties of the printed prosthesis were investigated using ANSYS software.

**Results:**

The yield strength of the titanium-alloy prosthesis was 950 ± 14 MPa (mean ± SD), and its ultimate strength was 1005 ± 26 MPa. The 3D finite element analyses revealed that the equivalent stress distribution of each prosthesis was unifrom. The symmetry and reconstruction quality contour of the repaired chest wall was satisfactory. No rejection or infection occurred during the 6-month follow-up period.

**Conclusion:**

Chest-wall reconstruction with a customized titanium-alloy prosthesis is a reliable technique for repairing bony defects.

## Background

Customized chest implants are widely used in the repair of chest-wall bony defects. The fixing plate is the most important and complicated component, and an inappropriate prosthesis might eventually lead to repair failure. Compared with prostheses manufactured by traditional methods, customized implants have the significant advantages of accurately restoring the appearance and normal function of the missing part. Optimizing the design and fabricating process of a customized implant markedly influences the strength of the prosthesis and the final outcome, and such techniques have been widely used by orthodontic and maxillofacial surgeons [1, 2].

This article presents two case reports on the utilization of three-dimensional (3D) image reconstruction technology, computer-aided design (CAD), and reverse-engineering mirroring to optimize the design of prostheses. Laser rapid-prototyping technology was used to produce titanium-alloy prostheses. The clinical results obtained in these two cases confirm the high quality of the chest-wall repairs.

## Methods

### Patient information

#### Case 1

**Fig. 1:**
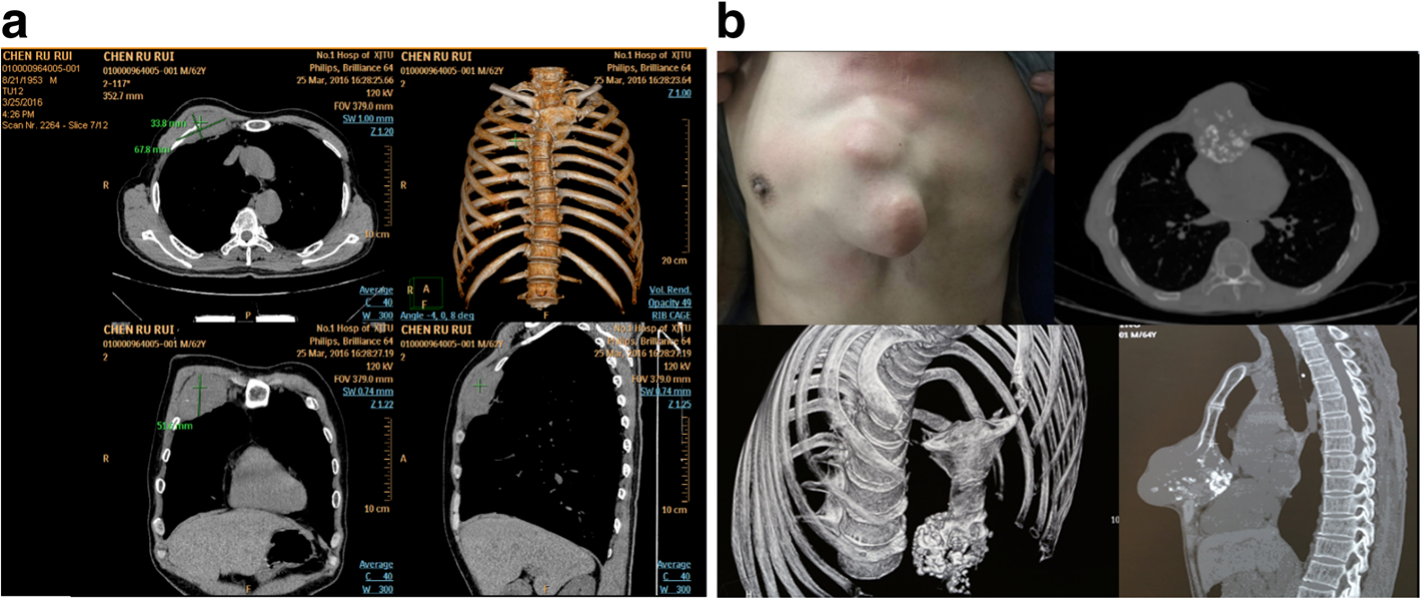
Preoperative images of the patients. **a** In case 1, the tumor protruded from the chest wall with destruction of the second and third ribs, and invaded the upper edge of the fourth rib. **b** In case 2, the tumor had destroyed infrasternal bone and protruded from the chest wall

A 62-year-old male was admitted for an anterior chest-wall mass. Chest CT revealed a right lung mass with right anterior chest-wall invasion. The tumor protruded from the chest wall with destruction of the second and third ribs, and invaded the upper edge of the fourth rib (Fig. 1a). The pathological diagnosis of a biopsy specimen was squamous cell carcinoma. A PET/CT evaluation showed no metastasis in the hilar or mediastinal lymph nodes, and no distant metastases were revealed. The patient was planned for surgery including resection of the tumor and ribs. The patient signed an informed-consent form and the surgery was approved by the appropriate ethics committee. Resection of the second and third ribs would lead to a large bone defect of the chest wall, and so an implantable rib prosthesis was designed for repairing the chest wall.

#### Case 2

A 64-year-old male was admitted for a chest-wall mass. Chest CT revealed a 5 × 5 × 5 cm^3^ mass in the middle of the chest wall. The tumor had destroyed sternal body and was considered to be a chondrosarcoma (Fig. 1b). No obvious contraindication or distant metastasis was revealed, and so the patient was assigned for surgery. The planned surgery included resection of the affected sternum, bilateral cartilage from the third to seventh ribs, and the related soft tissue. A sternum–rib prosthesis implantation was designed for reconstructing the chest wall.

### Design of the prosthesis

**Fig. 2:**
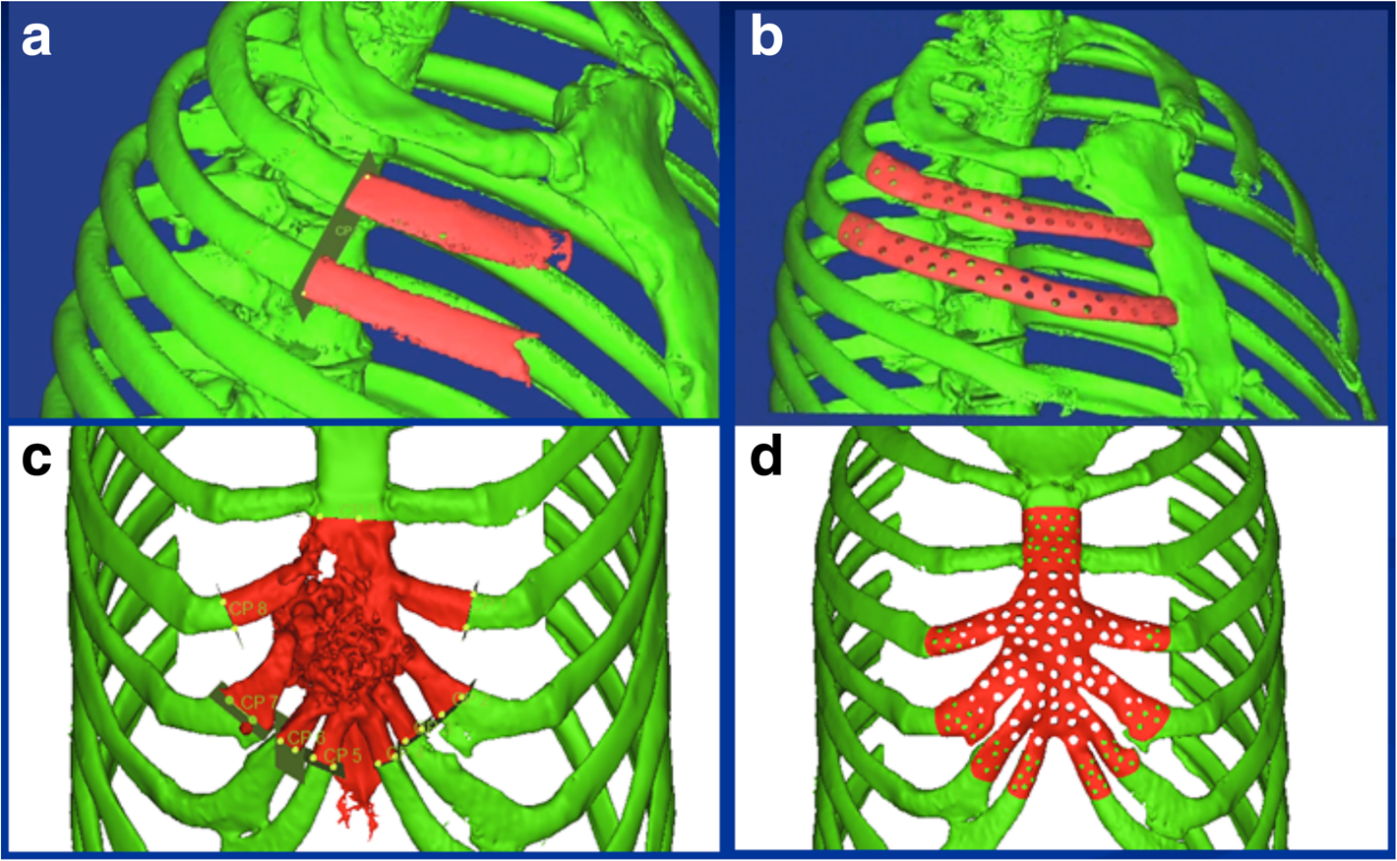
**a**, **c** The sternum and rib defects in the 3D reconstruction model for cases 1 and 2, respectively. **b**, **d** The corresponding customized prostheses designed by CAD and mirroring technology

A helical CT scan was acquired using a General Electric CT scanner at The First Affiliated Hospital of Xi’an Jiaotong University in Xi’an, China. This scan acquisition was performed with a 1.3-mm slice thickness, a slice reconstruction interval of 0.6 mm, and a 512 × 512 imaging matrix. The two-dimensional image slices obtained from the CT scans were imported into commercial Materialise Interactive Medical Image Control System (MIMICS) software (Materialise, Leuven, Belgium). After removing the soft tissue, the skeleton was visualized using a 3D display for diagnosing and evaluating the chest defect. Figure 2a and c show 3D images of the sternum–rib defects in cases 1 and 2, respectively.

Digisurf (3D–Family Corporation, Taiwan) software was used to design the surface shape of the prosthesis. MIMICS cannot directly interface the CT scanner with Digisurf software since the software can read only data obtained from a 3D laser scanner. Software called Point-Data-Processing was therefore developed in the Institute of Advanced Manufacturing Technology (Xi’an Jiaotong University) to convert the output from MIMICS in IGS format into the format readable by the Digisurf software for surface reconstruction. The CAD data for the prosthesis with corresponding resection templates were translated into the STL file format and imported into the prototyping machine to fabricate the physical prostheses (Fig. 2b and d). Alternatively, the stereolithographic apparatus (SLA) model prosthesis could be fitted on the 3D chest model to evaluate characteristics such as the symmetry and the accuracy of the surface fitting. Finally, the SLA prosthesis pattern was directly used in investment casting performed using Quick-Cast to produce the titanium-alloy prosthesis. Holes were made in the implant body after completing the CAD/CAM (computer-aided manufacturing) process in order to facilitate soft-tissue integration.

Since surgeries utilizes prefabricated titanium-alloy implants require a negative tumor margin, or ideally include more safety margin for some type of pathology, we estimate the resection margin and the dimensions of the implants based on preoperative 3D reconstruction. To ensure there is enough healthy bone to attach the implants, we also include a 2 cm redundant fixing area to accommodate the intraoperative alteration of surgical plan when designing the implants. This redundancy of fixing area greatly improves the safety of the surgery on both oncological and mechanical aspects.

### 3D finite element analysis

The CAD-generated virtual implants were positioned along the bony orbit according to surgical convention and prosthesis design considerations. The implants were virtually placed in the 3D viewing window, and then ANSYS (version 5.7, ANSYS, Pittsburgh, USA) finite element analysis software was used to analyze the stress distribution. The available bone at selected locations was evaluated in coronal, axial, and sagittal views, and implant positions were adjusted as needed while taking into account the prosthesis design requirements.

In this study we assumed that all of the materials were continuous, homogeneous, and isotropic linearly elastic. The material properties are listed in Table 1.

**Table 1:** Elastic modulus and Poisson’s ratio of the study materials

Material	Elastic modulus (MPa)	Poisson’s ratio
Cortical bone	13,800	0.26
Cancellous bone	345	0.31
Titanium-alloy	110,000	0.3

### Manufacturing the prosthesis

**Fig. 3:**
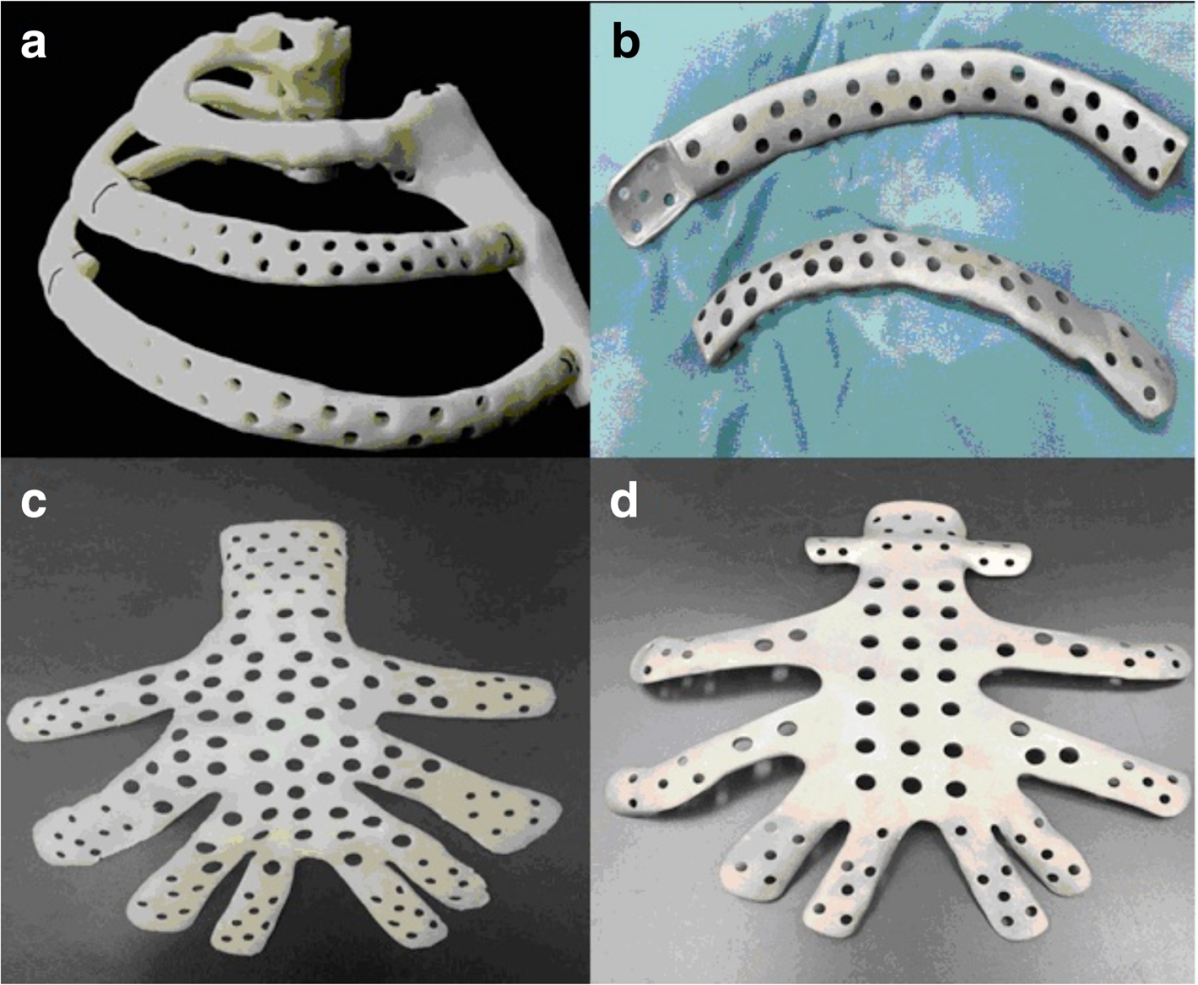
**a**, **c** Chest reference models for cases 1 and 2, respectively. **b**, **d** The corresponding 3D printed titanium-alloy prostheses

The 3D model of the patient’s chest was printed from the STL data of the patient’s skeletal anatomy (ZPrinter® 310 Plus, Z Corporation, Cambridge, MA, USA). The model was printed using high-performance composite powder and binder with a layer thickness of 0.102 mm. The surgical guide was test fitted on the 3D–printed model conforming to the patient’s anatomy to ensure a precise fit in the area in which the implant was to be placed surgically (Fig. 3a and c). The locations of the drilling sites were assessed by clinicians to ensure that they adequately represented the surgical plan before proceeding to surgery. Finally, the 3D data of the prosthesis was imported into the printing device to produce the titanium-alloy prosthesis, this step usually takes 3 work days. After mechanical polishing and sandblasting, the titanium-alloy prosthesis appeared silver white with a rough granular surface. No microscale defects such as scratches, cracks, burrs, or spiral edges were found in the inspection (Fig. 3b and d).

### Mechanical properties of the 3D–printed titanium-alloy prosthesis

The titanium-alloy prosthesis was fabricated by a laser rapid-prototyping system (EOS, Munich, Germany) from the raw material of spherical particles of titanium alloy with a diameter of 20–65 μm (Ti6Al4V, UNS R56400). The entire process was carried out in an environment protected using 99.999% argon to prevent the oxidation of titanium-alloy powder. In order to test the mechanical properties of titanium-alloy materials obtained by laser rapid prototyping, standard tensile specimens and three-point-bending specimens were prepared by the EOS system. The yield strength and ultimate strength of the formed titanium alloy were measured by SANS material testing machine (MTS Shenzhen, China) according to the test standard and environment temperature designated by the Ministry of Industry and Information Technology (GB 6892). The mechanical properties of the titanium-alloy prosthesis is listed in Table 2.

**Table 2:** Mechanical properties of the titanium-alloy prosthesis compared with industry standards

	Tensile strength (MPa)	Specified nonproportional extension strength, RP_0.2_ (MPa)	Elongation rate (%)
3D printed titanium-alloy prosthesis	1002	819	13.3
Pharmaceutical industry standards from YY0117.1–2005	≥860	≥780	≥10

Note: The YY0117.1–2005 pharmaceutical industry standards are entitled “Surgical implants: bone prosthesis forging, casting -Ti6Al4V titanium alloy forgings”

### Titanium-alloy rib prosthesis implantation and postoperative follow-up

#### Case 1

In the first case, the second to fourth ribs were resected along with the upper lobe of the right lung. The seventh and tenth groups of lymph nodes were also dissected to allow pathology analysis. After en-bloc resection of the tumor-bearing area, the measured defect area was 14×20 cm^2^. After disinfection and sterilization, the rib prosthesis was fixed with steel wire. Frozen section pathology of the resected edge is sent and came back negative. The skin incision was closed after the prosthesis was covered with a flap of the pectoralis major muscle. A chest drainage tube was routinely placed, and an elastic bandage was fixed with pressure. The operating time was 120 min and involved a minimal amount of blood loss. The postoperative pathology analysis showed squamous cell carcinoma of the right upper lung infiltrating the ribs. No lymph node metastasis was detected.

#### Case 2

In the second case, the prosthesis is designed to include a 2 cm error margin above the line of bone destruction. The sternum was resected below the 3rd sternal costal joints, the lateral border contains 3–7 costal cartilages, and a 1 cm margin above the intended resection line is also included due to unsatisfactory gross margin. The sternal prosthesis was fixed with the broken ends of the ribs and the rest of the sternum using screws. A flap of the rectus abdominis muscle was created and transferred to cover the prosthesis before closing the skin incision. Two drainage tubes were placed. The postoperative pathology analysis showed grade II chondrosarcoma with negative margins.

The integrity of the fixation was examined on postoperative day 1 in a chest x-ray, and a CT 3D reconstruction of the chest was reviewed 1 month after the surgery.

## Results

### 3D finite element analysis of the customized prosthesis

**Fig. 4:**
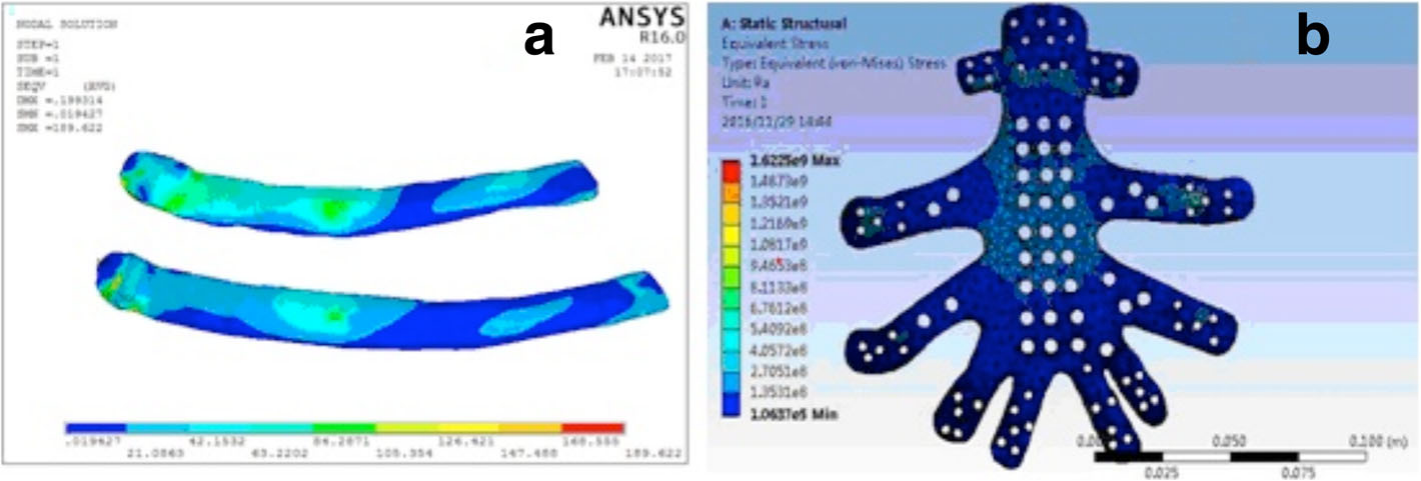
**a**, **b** Results of 3D finite element analyses of the prostheses for cases 1 and 2, respectively (stress unit: MPa)

A rapid biomechanical analysis was carried out to evaluate the stress distribution of each prosthesis. As shown in Fig. 4, the equivalent stress distribution of each prosthesis was uniform.

### Mechanical properties of the 3D titanium-alloy prosthesis

The yield strength of the 3D titanium-alloy rib prosthesis was 950 ± 14 MPa (mean ± SD), and its ultimate strength was 1005 ± 26 MPa. Sandblasting was applied to increase the surface roughness and thereby improve the connection between the prosthesis and soft tissue.

### Recovery and follow-up of rib prosthesis implantation

**Fig. 5:**
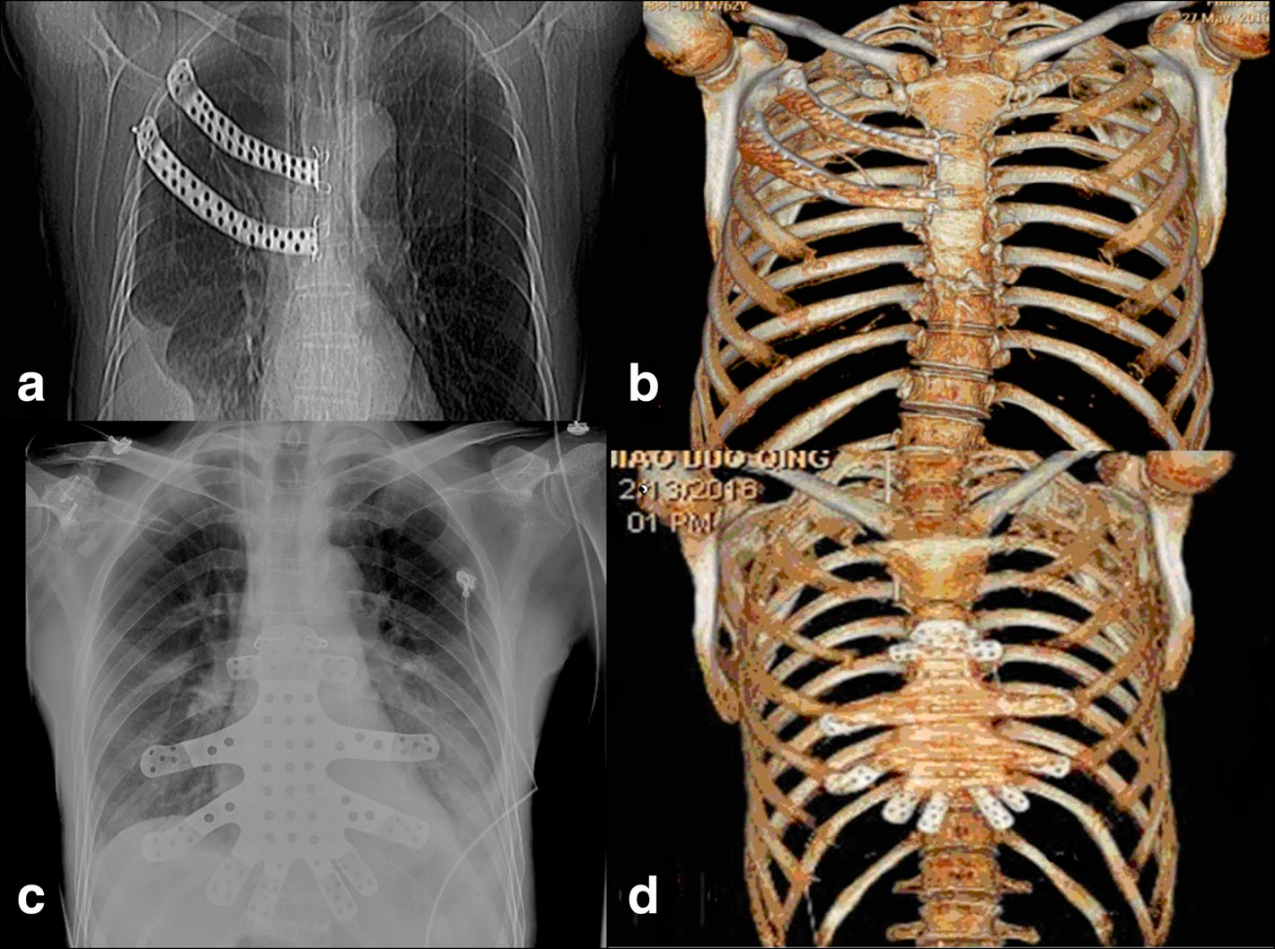
**a**, **c** Postoperative X-ray images showing that the prostheses fit well in the chest. **b**, **d** 3D reconstructions of CT data showing that the prostheses were well fixed in the chest

The radiological and clinical results obtained after reconstruction of the chest defects were ideal in the two patients. The customized prefabricated prostheses fit the chest defects well in both patients, and no adjustments were needed during the surgery. These features shortened the operating time. Rigid fixation of both implants was achieved using screws. Complications were not observed during the 6-month follow-up period. X-ray examinations showed no loosening of the prosthesis and no deformity of the chest (Fig. 5a and c). The 3D reconstruction of CT data showed that each prosthesis was well fixed and surrounded by fibrous tissue around the periosteum (Fig. 5b and d).

## Discussion

Large chest-wall defects can be caused by sternum or rib dysplasia, congenital malformation, trauma, and primary or secondary tumor lesions, and can have devastating functional and cosmetic consequences [3, 4]. Several reconstruction techniques such as autologous tissue transfers and alloplastic materials have been applied for many years, but reconstructing extensive defects with autologous bone is restricted by the amount of available donor bone, difficulty in 3D contouring, poor tissue tolerance and acceptance, and risk of long-term pain syndrome or discomfort [5]. Most thoracic surgeons agree that total or subtotal sternum resection or resecting more than three or four ribs increases the likelihood of chest-wall instability, and therefore is the typical indication for chest-wall reconstruction and the use of synthetic materials [6]. The use of alloplastic materials such as Marlex® mesh, bone cement, Prolene® mesh, Prolene® mesh composite bone cement, a sandwich complex, stainless steel net, titanium mesh, titanium plate, or plexiglass is more widely adopted [7], although commercially available materials are generally more cost-efficient but this is associated with risks including plate exposure, plate-screw fracture, screw loosening, infection, and poor cosmetic and functional restoration. It is also worth to point out that the cost of the custom prosthesis is not that high: $1200 for two ribs and $1300 for the sternum, depending on the weight of the prosthesis (how much alloys used) and complexity of the design.

Creating a 3D model of bone structures extracted from CT image data allows not only for precise prosthesis design but also provides adequate visualization of the defect for preoperative surgical evaluation and thorough planning of the repair. Few studies have focused on applying this technique to repairing chest defects. The two cases we report here have demonstrated the efficacy and accuracy of using combined technologies of 3D CT data and a stereolithography produced model for tumor resection guidance and defect reconstruction. The implantation of a titanium-alloy prosthesis covered with a flap of the pectoralis major muscle completely restored the normal chest appearance. The preoperative preparation, symmetry, and precise fit obtained during implant evaluation were greatly facilitated by using a physical model generated by rapid prototyping. This approach shortened the operating time and allowed for potential intraoperative errors to be modified preoperatively and thus avoided having to address them during the actual surgery. No complications occurred during the early postoperative period, other than slight chest-wall floating in case 2.

Until now most implants have been manually shaped intraoperatively at the surgical site [8]. However, modifying an implant intraoperatively is difficult. Furthermore, intraoperative modeling is time-consuming and is associated with unsatisfactory accuracy, sometimes even leading to more-invasive surgery or repair failure [9]. The intraoperative adaptation of the prosthesis can be avoided when using prefabricated customized titanium-alloy implants, and a CAD/CAM system can be used to design and fabricate prostheses with very complex 3D shapes. Moreover, CAD-based prosthesis modeling can avoid indirect manual manipulation on a life-size model. The application of prefabricated titanium-alloy implants in the two patients reported here has demonstrated that the prosthesis accuracy and the symmetry of the chest contour can be greatly improved.

## Conclusion

This study has shown that using a computer-generated model makes it possible to fabricate a custom implant that very accurately represents an anatomical defect. In addition to eliminating intraoperative errors and achieving a precise fit and high stability after screw fixation, the use of these techniques also leads to (i) better titanium-alloy bone and joint fixation with the use of titanium alloy, (ii) reduced occurrence of postoperative chest-wall floating and paroxysmal breathing, (iii) better tissue compatibility, fewer foreign-body reactions, and lower susceptible to infection, and (iv) overall better physiological and morphological contour of the chest. From the experience gained in the two present cases we can safely conclude that rapid-prototyping and CAD modeling can dramatically improved the presurgical planning workflow, with the subsequent fabrication process achieving sufficient precision after immediate reconstruction.

## Abbreviations

3D: Three-dimensional
CAD: Computer-aided design
CAM: Computer-aided manufacturing
MIMICS: Materialise interactive medical image control system
SLA: Stereolithographic apparatus
